# Integrative Analysis of the Metabolome and Transcriptome Reveals the Mechanism Underlying Differences in Skin Development Between the New Zealand Rabbit and the Rex Rabbit

**DOI:** 10.3390/ani16172746

**Published:** 2026-09-02

**Authors:** Bohao Zhao, Jiawei Cai, Ziyang Bai, Ting Chen, Aoyun Fan, Xinyu Cao, Yang Chen, Xinsheng Wu

**Affiliations:** 1 College of Animal Science and Technology, Yangzhou University, Yangzhou 225009, China; bhzhao@yzu.edu.cn (B.Z.); cjwcjw520800@163.com (J.C.); 19551508879@163.com (Z.B.); 17637894476@163.com (A.F.); 18020421120@163.com (X.C.); yangc@yzu.edu.cn (Y.C.); 2 Zhejiang Lingfei Rabbit Industry Co., Ltd., Cixi 315311, China; 13968287882@163.com; 3 Joint International Research Laboratory of Agriculture & Agri-Product Safety, Yangzhou University, Yangzhou 225009, China

**Keywords:** transcriptomics, metabolomics, rabbit, fur quality, skin development

## Abstract

Rex rabbit fur is a high-value material in the textile industry; however, the integrated metabolomic and transcriptomic mechanisms underlying differences in fur traits between fur-type Rex rabbits and meat-type New Zealand White rabbits remain incompletely understood. In this study, 40 rabbits (20 per group) raised under identical feeding conditions were used to evaluate fur quality traits. Dorsal skin samples were collected for metabolomic and transcriptomic analyses. The results showed that Rex rabbits had significantly lower fibre length and fur thickness than New Zealand White rabbits (*p* < 0.01). Metabolomic analysis identified 267 DMs, mainly comprising lipids and organic acids. These metabolites were enriched in butanoate metabolism, steroid hormone biosynthesis, cysteine and methionine metabolism, and the citrate cycle. Key metabolites included histamine, L-arginine, creatine, and indoxyl. Transcriptomic analysis identified 895 DEGs, enriched in MAPK, cAMP, and HIF-1 signaling pathways, including keratin family genes (*KRT34*, *KRT84*, *KRT82*), *FGFR4*, *EGF*, *BMP5*, and *DKK2*. Integrated analysis identified 211 DEGs and 97 DMs co-enriched in 103 pathways, including HIF-1, cAMP, and AMPK signaling pathways. These findings reveal a gene–metabolite regulatory network involved in skin and hair follicle development, provide molecular markers for improving rabbit fur quality, and enhance understanding of the genetic and metabolic basis of mammalian hair follicle development.

## Simple Summary

The Rex rabbit is a representative fur-type breed, yet the molecular mechanisms underlying its distinct fur characteristics relative to meat-type rabbit breeds remain incompletely understood. In this study, fur quality traits were evaluated in Rex and New Zealand White rabbits, followed by integrated metabolomic and transcriptomic profiling of dorsal skin tissues. Rex rabbits showed significantly shorter fibre length and reduced fur thickness compared with New Zealand White rabbits. A total of 267 differential metabolites and 895 differentially expressed genes were identified, with joint enrichment in pathways associated with hair follicle development, including HIF-1, cAMP, and AMPK signaling. Histamine, L-arginine, keratin family genes, *BMP5*, and *DKK2* were identified as key molecular components associated with fur development. These results provide insights into the coordinated gene–metabolite regulatory mechanisms underlying rabbit skin and hair follicle development and identify potential molecular markers for improving fur quality through selective breeding.

## 1. Introduction

The Rex (RX) rabbit is a well-known fur-producing breed with superior coat characteristics, which are highly valued for their lightness, softness, color, thermal insulation, and aesthetic quality [1]. In comparison with the meat-type New Zealand White (NZW) rabbit, RX rabbits show distinct differences in fur quality. Previous studies have reported differences in skin histological structure between NZW and RX rabbits, including greater total skin thickness and thicker hypodermal layers in NZW rabbits than in RX rabbits [2,3,4]. Therefore, NZW and RX rabbits provide an ideal comparative model for elucidating the relationship between skin development and fur trait formation.

Hair follicles (HFs) are complex mini-organs that regulate the growth and development of mammalian hair [5]. HF morphology, growth, and development directly determine wool length, density, fibre fineness, and overall coat quality in animals [6,7]. Several studies have investigated the molecular mechanisms regulating skin and HF development in RX rabbits. Genes such as *BMP2*, *EGF*, *IGF-I*, *β-catenin*, and members of the *TGF-β* family show differential expression in RX dorsal skin from birth to 8 weeks of age [8]. Signaling pathways, including Wnt/β-catenin, TGF-β, and BMP, have been reported to regulate HF development in RX rabbits [9]. AMPK signaling serves as a key energy-sensing pathway that regulates the balance between lipid synthesis and breakdown, thereby supporting normal HF development and fur formation in rabbits [10]. In rabbit dermal papilla cells, HIF-1α signaling promotes HF growth through VEGF-mediated perifollicular angiogenesis, whereas suppression of this pathway impairs HF development [11]. To explore the roles of coding and non-coding RNAs in HF cycling in RX rabbits, differentially expressed mRNAs were found to be enriched in PI3K-Akt and Wnt signaling pathways [12]. Breed-specific variation in skin and HF development has also been investigated; for example, m6A RNA methylation profiling between RX and Hycole rabbits revealed enrichment of cAMP, Hippo, MAPK, and Wnt signaling pathways associated with HF development [13]. Furthermore, whole-genome resequencing has identified key genes associated with long wool traits in Angora, RX, and NZW rabbits [3]. However, a systematic comparison of gene expression profiles between RX (fur-type) and NZW (meat-type) rabbits remains insufficient.

Integrative transcriptomic and metabolomic analyses have been applied to elucidate the molecular mechanisms underlying wool fibre growth and hair development. The genes associated with wool fibre diameter and lipid metabolites related to wool performance have been investigated in fine wool sheep using non-targeted metabolomics and transcriptomics [14]. Co-enriched metabolic pathways, including the FOXO signaling pathway, protein digestion and absorption, and arginine and proline metabolism, have been identified through integrative transcriptomic and metabolomic analyses to explain melatonin-induced cashmere growth [15]. Integrated transcriptomic and metabolomic profiling has also revealed that psychological stress alters gene expression and metabolite profiles associated with hair loss in murine models, with metabolic pathways such as arachidonic acid metabolism, glutathione metabolism, and purine metabolism being implicated [16]. Furthermore, previous studies have reported significant differences in amino acid profiles, lipid metabolites, fatty acid composition, and volatile compound content between NZW and RX rabbits [17,18]. However, the integrated regulatory mechanisms linking transcriptomic and metabolomic changes underlying fur trait differences between these two breeds remain largely unexplored, and elucidating these mechanisms is essential for improving breeding strategies aimed at fur quality enhancement.

The aim of this study is to identify differential metabolites (DMs and DEGs) in skin tissues of RX and NZW rabbits at the fully grown stage and to characterize their associated biological functions through integrated untargeted metabolomics and transcriptomics. The study further aimed to elucidate the molecular mechanisms underlying the distinct fur phenotypes between the two breeds, with particular emphasis on HIF-1 and AMPK signaling pathways, amino acid biosynthesis, and lipid metabolism. This study provides a theoretical basis for molecular breeding of rabbit fur quality traits and offers a reference for improving wool growth in wool-producing animals.

## 2. Materials and Methods

### 2.1. Experimental Animal

Rabbits were obtained from Zhejiang Province Lingfei Rabbit Industry Co., Ltd. (Cixi, China). A total of 40 healthy, fully grown 13-week-old rabbits were selected for the study [19,20], comprising RX rabbits (*n* = 20) and NZW rabbits (*n* = 20). Ten rabbits from each group were randomly selected for metabolomic profiling, and 6 of these rabbits per group were further selected for paired RNA sequencing using mid-dorsal skin samples obtained from the same individuals. The groups included equal numbers of males and females and were maintained under identical feeding and housing conditions. For sample collection, rabbits were anesthetized via intravenous administration of Zoletil-50 (Virbac, Carros, France) at a dose of 8 mg/kg body weight through the ear vein. Following euthanasia, fur samples were collected for the assessment of wool length, fur thickness, and fur area. Approximately 1 cm^2^ of mid-dorsal skin was excised for further RNA sequencing and metabolomic analysis. All experimental procedures were approved by the Animal Care and Use Committee of Yangzhou University, China (No. 202503017).

### 2.2. Fur Quality Index Determination

Wool fibre length was measured in the natural, unstrained state using a ruler. Fur thickness was determined at the dorsal region using a digital vernier caliper. Fur length was determined as the distance along the dorsal midline from the neck to the tail using a measuring tape, whereas fur width was measured as the left-to-right distance across the mid-dorsal region using the same method. Fur area (cm^2^) was calculated as fur length (cm) × fur width (cm).

### 2.3. Untargeted Metabolomics Analysis

Untargeted metabolomic profiling was performed on skin tissue samples collected from RX rabbits (R group, *n* = 10) and NZW rabbits (N group, *n* = 10). For each sample, 100 mg of dorsal skin tissue was pulverized under liquid nitrogen. The powder was resuspended in pre-cooled 80% methanol containing 0.1% formic acid and thoroughly vortexed. Samples were incubated on ice for 5 min, followed by centrifugation at 15,000× *g* at 4 °C for 5 min. The resulting supernatant was diluted with LC–MS-grade water to achieve a final methanol concentration of 53% and centrifuged again at 15,000× *g* and 4 °C for 10 min. The final supernatant was subjected to UHPLC–MS/MS analysis using a Vanquish UHPLC system (Thermo Fisher, Bremen, Germany) coupled to an Orbitrap Q Exactive™ HF mass spectrometer (Thermo Fisher, Bremen, Germany), equipped with a Hypesil Gold column (100 × 2.1 mm, 1.9 μm) and a 17 min gradient elution program at a flow rate of 0.2 mL/min.

For positive polarity mode, the mobile phases were eluent A (0.1% FA in water) and eluent B (methanol). For negative polarity mode, the mobile phases were eluent A (5 mM ammonium acetate, pH 9.0) and eluent B (methanol). The gradient program was as follows: 2% B for 1.5 min; 2–100% B over 12.0 min; 100% B for 14.0 min; 100–2% B at 14.1 min; and 2% B until 17 min. The Q Exactive™ HF mass spectrometer was operated in electrospray ionization positive/negative switching mode, with a spray voltage of 3.2 kV, capillary temperature of 320 °C, sheath gas flow rate of 40 arb, and auxiliary gas flow rate of 10 arb.

Raw data were processed using Compound Discoverer 3.1 (CD3.1; Thermo Fisher Scientific), and Data analysis was performed using R software (version 4.4.0). Metabolite annotation was carried out using the KEGG, HMDB, and LIPID MAPS databases. Metabolites meeting the criteria of fold change (FC) ≥ 1.2 or ≤1/1.2, variable importance in projection (VIP) > 1 from the OPLS-DA model, and *p* < 0.05 were defined as differentially expressed metabolites (DMs). Raw data were deposited in the OMIX database of the China National Center for Bioinformation (CNCB) under accession number OMIX017024. Functional enrichment analysis of DMs and associated metabolic pathways was performed using the Kyoto Encyclopedia of Genes and Genomes (KEGG) database.

### 2.4. RNA Sequencing

For transcriptome analysis, dorsal skin samples were collected from RX rabbits (R group, *n* = 6) and NZW rabbits (N group, *n* = 6). Following quality assessment of RNA purity and concentration, RNA-seq libraries were constructed, and high-throughput sequencing was performed on the Illumina NovaSeq 6000 platform to generate raw reads. After quality filtering, clean reads were aligned to the *Oryctolagus cuniculus* reference genome (OryCun2.0) using HISAT2. Differential expression analysis was performed using the DESeq2 R package, with thresholds set at |log_2_FC| ≥ 1 and *False Discovery Rate* (*FDR*) < 0.05 to identify DEGs. All raw sequencing data have been deposited in the Genome Sequence Archive (GSA) of the CNCB under accession number CRA043744. Functional enrichment analysis of DEGs was performed using Gene Ontology (GO) and Kyoto KEGG pathway analyses to investigate their biological roles.

### 2.5. Integrative Analysis of Transcriptome and Metabolome

DMs and DEGs identified from transcriptome sequencing and untargeted metabolomics assays were used for integrative analysis using six paired rabbit samples, respectively. *Pearson* correlation analysis was performed to evaluate pairwise correlations among DMs, DEGs, and key fur quality traits. Gene–metabolite pairs with an absolute correlation coefficient *|r|* > 0.90 were selected as highly correlated interactions. These pairs were visualized as a correlation chord diagram using the R package *circlize* (version 0.4.15). Furthermore, KEGG pathway information jointly annotated by DEGs and DMs was integrated to construct an interactive metabolic network, which was visualized using the R package *ggnetwork* (version 0.5.10).

### 2.6. Total RNA Isolation and Real-Time Quantitative PCR

Total RNA was isolated from skin samples using the SteadyPure RNA Kit (Accurate Biology, Changsha, China), and cDNA was synthesized using the Evo M-MLV Reverse Transcription Kit (Accurate Biology, Changsha, China). Quantitative real-time PCR (RT-qPCR) was performed on a QuantStudio^®^ 5 Real-Time PCR System using the SYBR Green Premix Pro Taq HS qPCR Kit (Accurate Biology, Changsha, China) by following the manufacturer’s protocol. Relative gene expression levels were normalized to GAPDH and calculated using the 2^−ΔΔCt^ method. Primer sequences are provided in Supplementary Table S1.

### 2.7. Statistical Analysis

Statistical analyses were performed using SPSS 27.0 statistical software (IBM Corp., Armonk, NY, USA). Data are presented as mean ± standard deviation. Differences between the two groups were analyzed using *Student’s t*-test. Value of *p* < 0.05 was considered statistically significant.

## 3. Results

### 3.1. Phenotypic Differences Between RX Rabbits and NZW Rabbits

**Figure 1:**
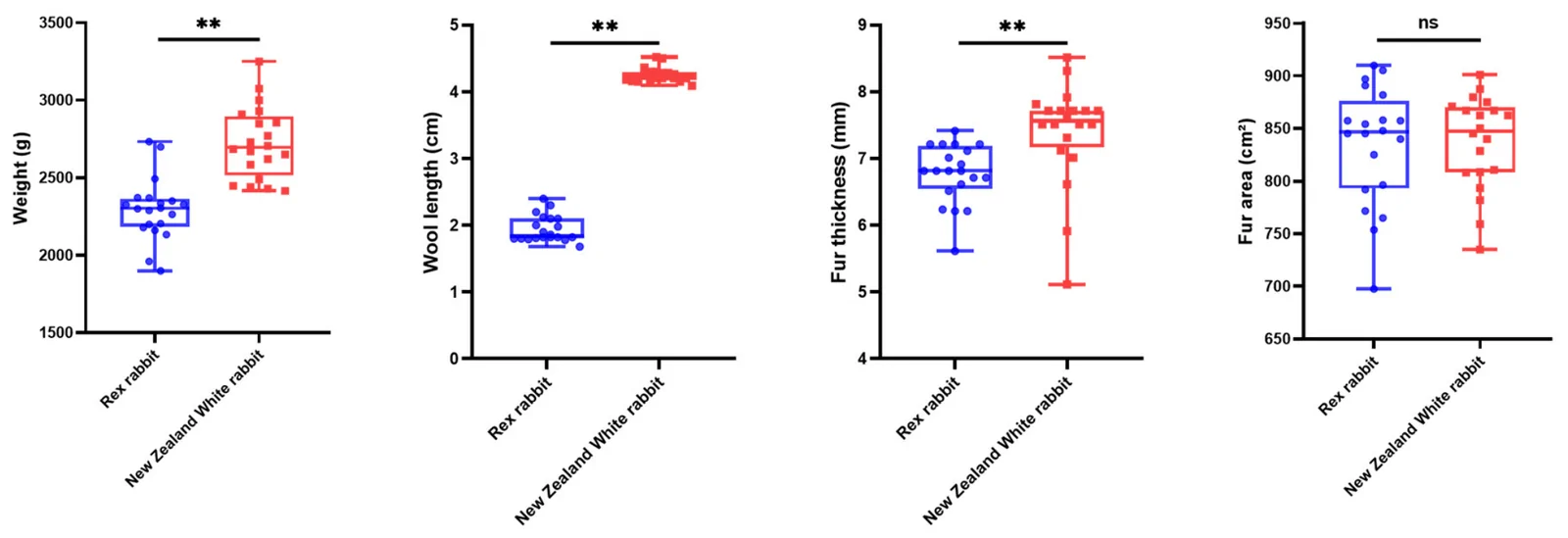
Comparison of weight, fibre length, fur thickness and fur area between RX rabbits and NZW rabbits. ** *p* < 0.01.

At baseline, body weight was significantly higher in NZW rabbits than in RX rabbits (*p* < 0.01). Further analysis of fur traits revealed that fibre length and fur thickness were significantly lower in RX rabbits compared with NZW rabbits (*p* < 0.01), whereas no significant difference in fur area was observed between the two groups (Figure 1).

### 3.2. Differential Metabolite Profiling in Skin Tissues of RX and NZW Rabbits

**Figure 2:**
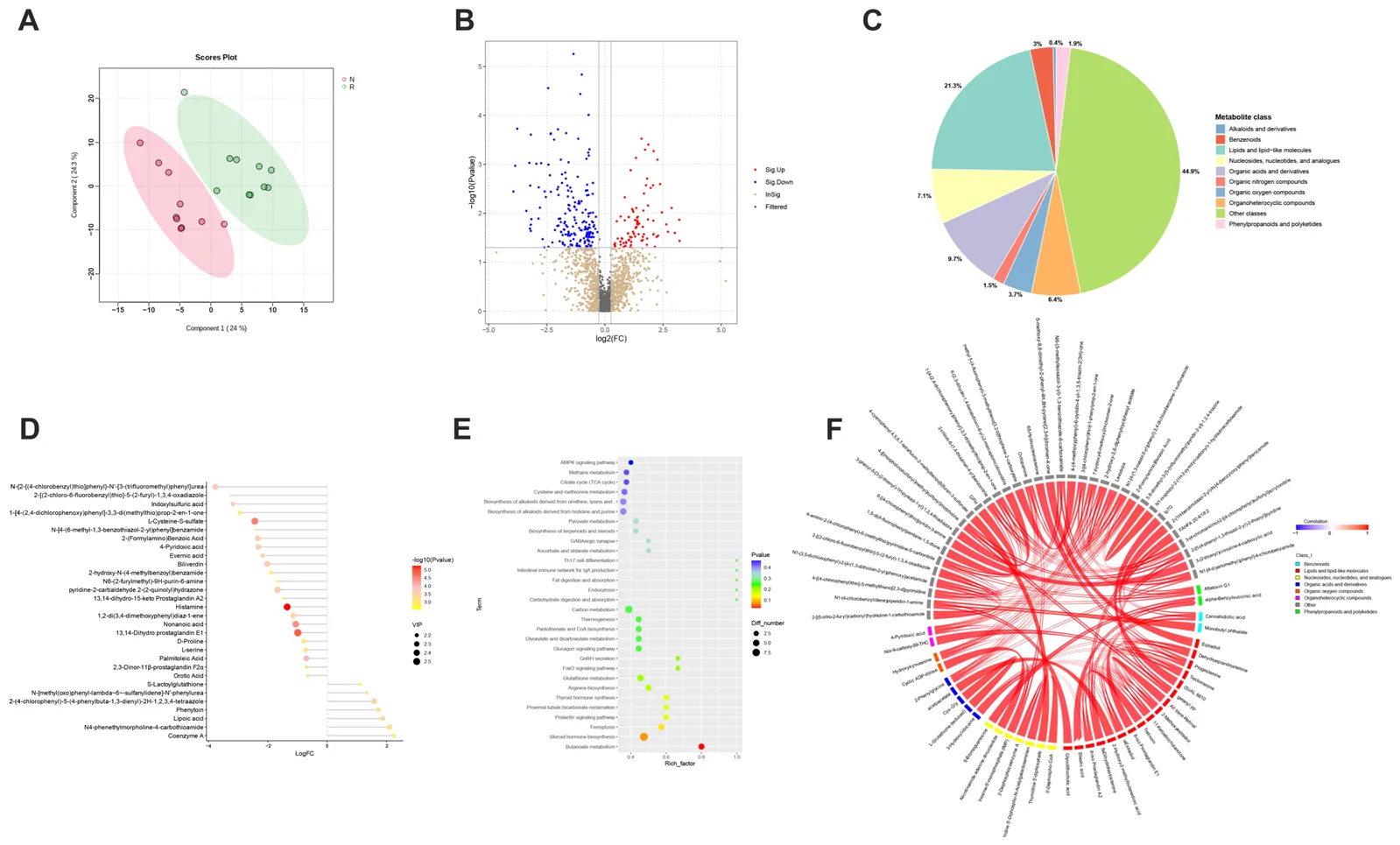
Metabolomic profiling and differential analysis of dorsal skin tissues from NZW and RX rabbits. (**A**) OPLS-DA score plot showing the separation of metabolic profiles between NZW and RX rabbits. (**B**) Volcano plot showing the distribution of DMs identified between NZW and RX rabbits. (**C**) Classification of DMs according to chemical categories. (**D**) Top 30 DMs identified between NZW and RX rabbits. (**E**) KEGG pathway enrichment analysis of DMs. (**F**) Correlation analysis showing the associations among identified metabolites.

Metabolites in dorsal skin tissues from RX and NZW rabbits were profiled and classified using LC-MS-based metabolomics. OPLS-DA analysis revealed clear separation between the two groups, indicating distinct metabolic profiles (Figure 2A). A total of 267 DMs were identified in RX compared with NZW, including 84 up-regulated and 183 down-regulated metabolites in the RX group (Figure 2B; Table S2). These DMs included histamine, L-arginine, pyridoxine, creatine, and testosterone. Chemical classification showed that lipids and lipid-like molecules constituted the largest category, followed by organic acids and derivatives and nucleosides, nucleotides, and analogues (Figure 2C). The top 30 DMs are presented in Figure 2D, including histamine, L-cysteine-S-sulfate, nonanoic acid, indoxylsulfuric acid, and 13,14-dihydro prostaglandin E1.

Several identified metabolites have established roles in skin and HF biology. Histamine released by keratinocytes regulates skin and HF physiology [21]. Cysteine contributes to keratin synthesis in keratinocytes and promotes wool production through modulation of cellular iron homeostasis [22]. L-arginine serves as an important substrate for keratin synthesis [23]. KEGG pathway enrichment analysis indicated that DMs were significantly associated with butanoate metabolism, steroid hormone biosynthesis, cysteine and methionine metabolism, ferroptosis, citrate cycle (TCA cycle), AMPK signaling pathway, and FoxO signaling pathway (Figure 2E). Metabolite correlation analysis further revealed strong interactions among DMs across multiple metabolite classes, including 17 lipids and lipid-like molecules, 7 nucleosides, nucleotides and analogues, 5 organic acids and derivatives, 2 benzenoids, 2 organic oxygen compounds, 2 organoheterocyclic compounds, 2 phenylpropanoids and polyketides, and 37 metabolites classified into other categories, indicating a coordinated metabolic regulatory network (Figure 2F).

### 3.3. Differential Gene Expression Profiling in Skin Tissues of RX and NZW Rabbits

**Figure 3:**
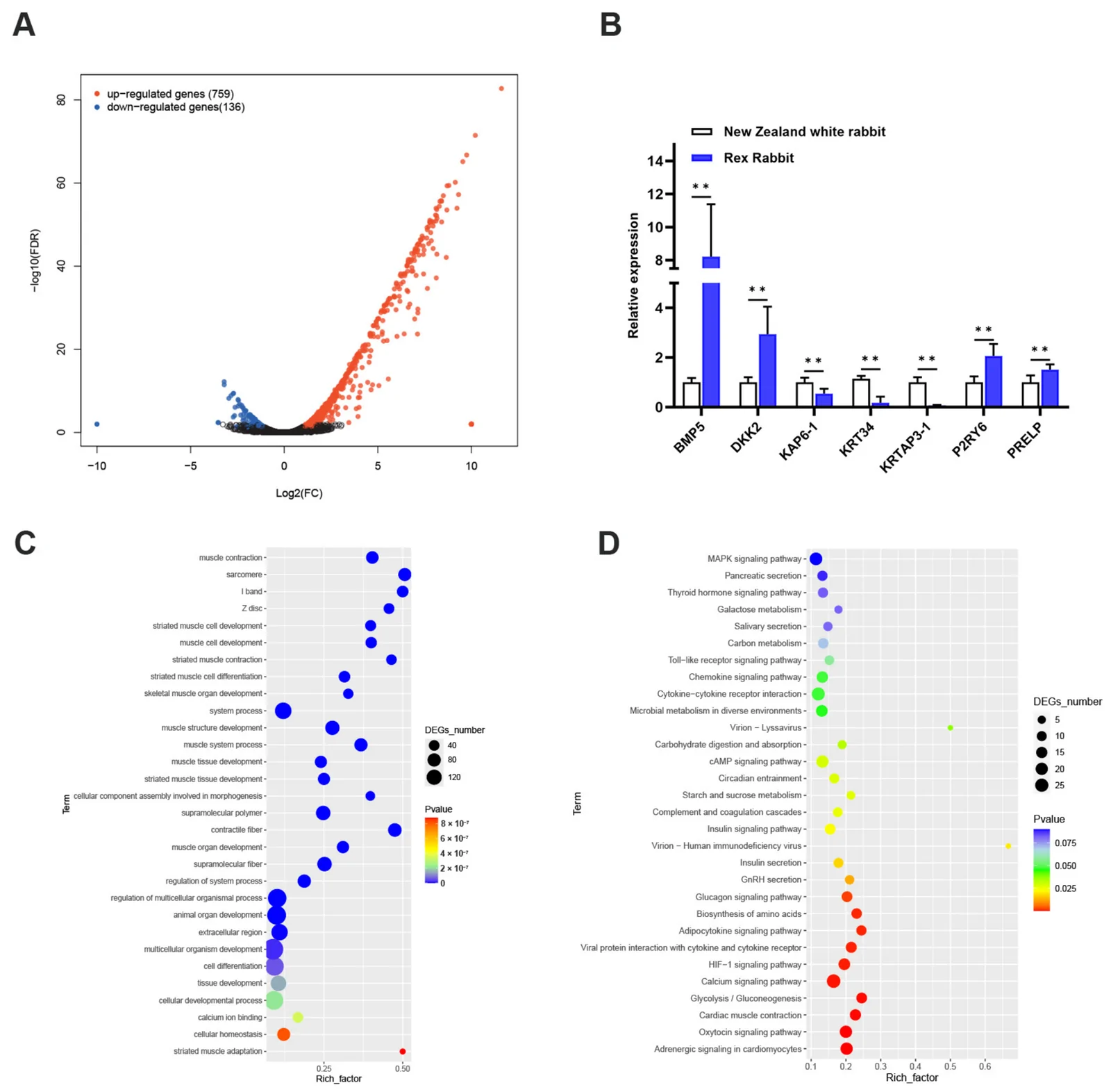
Transcriptomic analysis of DEGs in dorsal skin tissues from NZW and RX rabbits. (**A**) Identification of DEGs between NZW and RX rabbits. (**B**) Validation of DEG expression patterns by RT-qPCR analysis. (**C**) GO functional enrichment analysis of DEGs. (**D**) KEGG pathway enrichment analysis of DEGs. ** *p* < 0.01.

RNA-seq was performed on skin tissues from RX rabbits and NZW rabbits. Quality control and mapping statistics of the RNA-seq data indicated that the sequencing data were suitable for downstream analysis (Table S3). A total of 895 DEGs were identified, including 759 up-regulated and 136 down-regulated genes (Figure 3A; Table S4). For validation, seven DEGs (*BMP5*, *DKK2*, *KAP6-1*, *KRT34*, *KRTAP3-1*, *P2RY6*, and *PRELP*) were randomly selected for RT-qPCR analysis, and the expression patterns were consistent with the RNA-seq results (Figure 3B). GO enrichment analysis showed that DEGs were significantly associated with terms including regulation of system process, cellular homeostasis, and cell proliferation (Figure 3C). GO terms related to HF development, such as keratinization, animal organ development, and tissue development, were also significantly enriched. KEGG pathway enrichment analysis indicated that DEGs were mainly enriched in the MAPK, Toll-like receptor, cAMP, and HIF-1 signaling pathways (Figure 3D). Several pathways previously implicated in HF development, including MAPK, HIF-1, TGF-β, AMPK, and VEGF signaling pathways, were also enriched. Candidate DEGs associated with HF growth and development included *KRT34*, *KRT84*, *KRT82*, *KRTAP3-1*, *KRTAP6-1*, *FGFR4*, *EGF*, *BMP5*, and *DKK2*.

### 3.4. Integrated Analysis of Metabolome and Transcriptome

**Figure 4:**
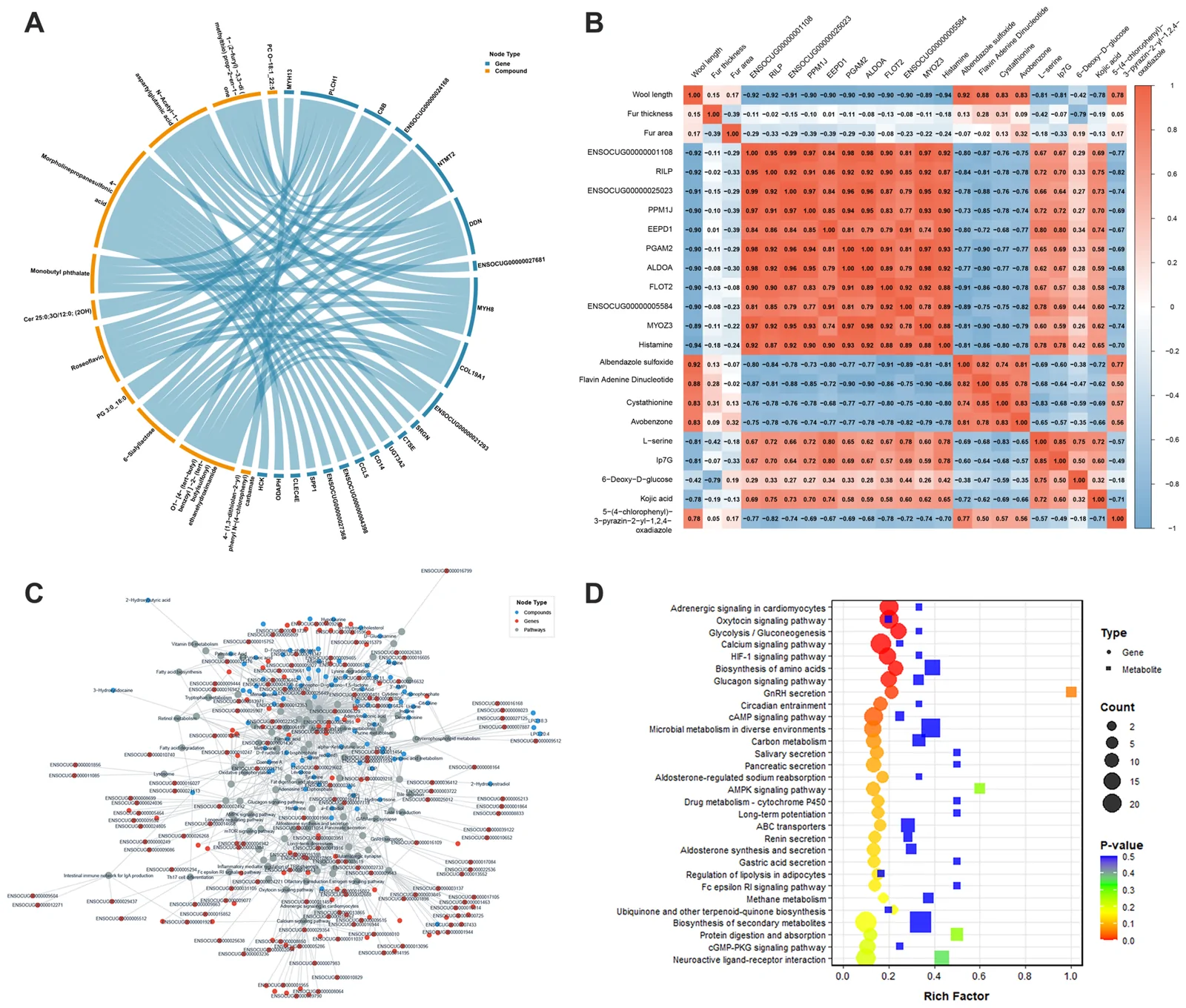
Integrative analysis of DEGs and DMs. (**A**) Correlation analysis of the top 50 DEG–DM pairs ranked by correlation coefficient. (**B**) Correlation heatmap showing associations among DMs, DEGs, fibre length, fur thickness, and fur area. (**C**) Interactive regulatory network constructed based on DEGs, DMs, and co-annotated KEGG signaling pathways. (**D**) Integrated KEGG pathway enrichment analysis of DEGs and DMs.

Integrated analysis was performed based on the expression profiles of DMs and DEGs between RX and NZW rabbits to elucidate regulatory differences in skin metabolism and gene expression. The results showed that the expression of genes including *MYH8*, *MYH13*, *PLCH1*, *COL19A1*, and *NTMT2* was significantly correlated with the abundance of metabolites such as 4-morpholinepropanesulfonic acid, 1-(2-furyl)-3,3-di(methylthio)prop-2-en-1-one, monobutyl phthalate, N-acetyl-1-aspartylglutamic acid, and roseoflavin (Figure 4A). To further characterize the relationships among DMs, DEGs, and key fur traits, a correlation heatmap was generated to visualize pairwise associations among DMs, DEGs, fibre length, fur thickness, and fur area (Figure 4B). Among the identified metabolites, histamine showed a significant positive correlation with *PGAM2* (*p* < 0.01) and a highly significant negative correlation with fibre length (*p* < 0.01). However, the absolute correlation coefficients between DEGs or DMs and fur thickness or fur area were all below 0.90, indicating that the molecular mechanisms underlying these two fur traits require further investigation. An integrated network of DEGs, DMs, and pathways was constructed, revealing that 211 DEGs and 97 DMs were jointly enriched across 103 metabolic pathways (Figure 4C). Among these, 85 DEGs associated with metabolic pathways showed strong correlations with 86 DMs, while 32 DEGs involved in the biosynthesis of secondary metabolites were correlated with 30 DMs. Furthermore, 12 DEGs enriched in the biosynthesis of amino acids showed associations with 11 DMs, and 5 DEGs involved in the biosynthesis of cofactors were correlated with 27 DMs. Furthermore, LDHA and LDHB in the HIF-1 signaling pathway showed strong associations with α-ketoglutaric acid. Genes *PRKAA*, *PRKAB*, and *PRKAG* in the AMPK signaling pathway were strongly correlated with metabolites such as adenosine 5′-diphosphate and L-glutamate. *PRKAA1* and *INSR*, involved in the mTOR signaling pathway, were associated with L-arginine. KEGG enrichment analysis of integrated DMs and DEGs indicated significant enrichment of calcium signaling, HIF-1 signaling, cAMP signaling, biosynthesis of amino acids, and AMPK signaling pathways (Figure 4D).

## 4. Discussion

Genetic improvement of fur quality remains an important objective in commercial RX rabbit breeding, and elucidating the molecular mechanisms underlying breed-specific fur phenotypes is relevant to both HF biology and molecular breeding strategies in the fur industry. RX rabbit fur is a globally valued raw material in the fur and textile industries owing to its softness, low fibre diameter, and distinctive appearance [24]. Previous studies have reported that wool length in RX rabbits is significantly shorter than in NZW rabbits [3,25] and that fur thickness in RX rabbits is lower compared with NZW rabbits [26,27]. These observations are consistent with the present findings, in which fibre length and fur thickness were significantly reduced in RX rabbits relative to NZW rabbits. No significant difference was observed in fur area between the two breeds, which aligns with earlier reports [2]. These results provide a systematic quantification of phenotypic variation in fur characteristics between the two rabbit breeds. However, the molecular mechanisms underlying differences in skin metabolites and gene expression between fur-type and meat-type rabbit breeds remain insufficiently characterized through integrated transcriptomic and metabolomic approaches.

Metabolomics has identified metabolites associated with the HF cycle, including amino acids and their derivatives, organic acids and their derivatives, nucleotides, and fatty acyls in cashmere goats [28]. Lipids and lipid-like molecules have also been implicated in regulating duck skin development during embryogenesis and are differentially expressed between coarse and fine cashmere skin in goats [29,30]. In this study, lipids and lipid-like molecules accounted for 21.35% of all DMs, representing the most abundant class, and mainly included estradiol, glycolithocholic acid, cholesteryl sulfate, and nonanoic acid. Lipids contribute to the formation of protective barriers within hair fibres, influence fibre strength and texture, and play important roles in hair formation and skin homeostasis [31,32]. The altered lipid metabolite profile observed in RX rabbits may be associated with their characteristic fur phenotype, consistent with previous evidence that lipid metabolism contributes to the regulation of wool fibre texture [14]. A total of 267 metabolites were differentially expressed between RX and NZW rabbit skin, including histamine, L-arginine, pyridoxine, creatine, and testosterone. Previous studies have reported that histamine released from keratinocytes modulates skin and HF physiology and may mediate responses to mechanical stimuli as well as contribute to dermatological disease pathogenesis [21,33]. L-arginine has been reported to be differentially expressed in cashmere goat skin during the transition from anagen to telogen [28]. Pyridoxine has been shown to enhance HF density in RX rabbits through activation of the PI3K/Akt, Wnt, and Notch signaling pathways [34]. The creatine kinase system provides energy to skin and HFs, and its dysregulation has been associated with skin injury and psoriasis progression [35]. Testosterone has been reported to increase sebaceous gland size and promote de novo sebaceous gland formation [36]. Furthermore, multiple metabolic pathways identified in this study, including butanoate metabolism, the TCA cycle, and cysteine and methionine metabolism, have been associated with HF growth and development. Butanoate metabolism has been implicated in the progression of hair loss [37]. Dysregulation of the TCA cycle may induce metabolic quiescence and relative nutrient deficiency in miniaturized HFs, contributing to follicular miniaturization in female pattern hair loss [38]. Cysteine promotes keratin synthesis in keratinocytes and can rescue iron deficiency-induced impairment of keratin expression through modulation of iron homeostasis, while dietary methionine supplementation improves wool production in Angora rabbits [22]. Methionine is essential for HF maintenance and growth and cannot be substituted by homocysteine to prevent alopecia [39]. Furthermore, RNA-seq analysis identified 895 DEGs between RX and NZW rabbit skin tissues, among which several genes associated with HF development were highlighted. Members of the KRTs and KAPs constitute major structural components of the hair shaft and are key determinants of hair fibre mechanical properties [40]. Hair keratin genes, including *KRT34*, *KRT84*, and *KRT82*, have been reported to regulate the hair cycle as well as hair growth and development [41,42,43], and were differentially expressed between the two rabbit groups in this study. The keratin-associated protein genes *KRTAP3-1* and *KRTAP6-1* were downregulated in the RX group. *KRTAP3-1* has previously been implicated in HF cycling and identified as a hub gene during the catagen phase [44], while *KRTAP6-1* expression has been reported to be negatively correlated with wool fibre diameter [45]. *BMP5* is highly expressed in mouse HF bulge regions and interfollicular epidermis, with dynamic expression during HF cyclic regeneration [46]. The deletion of *DKK2* has been shown to induce hair growth in mouse plantar skin and regulate skin and hair fate determination [47]. In this study, *BMP2* and *DKK2* were significantly upregulated in the RX group, suggesting potential inhibitory roles in skin and HF growth and development in rabbits. KEGG pathway enrichment analysis of DEGs further indicated significant involvement of signaling pathways related to HF biology, including MAPK, TLR, cAMP, and HIF-1 signaling pathways [48,49,50,51].

Integrated transcriptomic and metabolomic profiling revealed strong correlations between DEGs and DMs. Histamine showed a significant negative correlation with wool length and a positive correlation with *PGAM2*, a key regulator of glycolysis [52]. These findings suggest a potential regulatory relationship between histamine-associated signaling and glycolytic activity in relation to HF growth in rabbit skin. The relatively weak correlations observed between DEGs or DMs and fur thickness and fur area suggest that these complex polygenic traits may be influenced by multiple regulatory layers that cannot be adequately captured by simple linear transcript–metabolite associations. After integrated metabolomic and transcriptomic analysis, the results showed that DEGs and DMs were co-enriched in multiple key pathways, with 211 DEGs and 97 DMs jointly mapped to 103 pathways, including AMPK, cAMP, and HIF-1 signaling pathways. The AMPK signaling pathway has been reported to regulate dermal papilla cell proliferation and hair growth [53]. The cAMP signaling pathway is involved in the regulation of HF stem cell (HFSC) activation and the hair cycle [54]. Lipids may regulate cell proliferation and trichogenic gene expression in DPCs and modulate hair shaft formation through the HIF-1 pathway [55]. LDH activity has been associated with the telogen-to-anagen transition, with LDHA being enriched in quiescent HFSCs during the telogen phase [56]. *LDHA* and *LDHB* within the HIF-1 signaling pathway are also associated with α-ketoglutaric acid. α-Ketoglutaric acid has been shown to significantly promote DPC proliferation and contribute to HF development in RX rabbits [57]. The integrated DM–DEG regulatory network provides a framework for understanding breed-specific interactions between gene expression and metabolite profiles in RX and NZW rabbits. However, the present study identified associations between key DEGs and DMs but did not establish their causal roles in rabbit HF growth and development. Future studies incorporating molecular and functional experiments are warranted to validate these regulatory relationships and clarify their mechanistic roles in HF development.

## 5. Conclusions

In conclusion, clear phenotypic and molecular differences in fur traits exist between RX and NZW rabbits. These differences arise from coordinated changes in gene expression and metabolite profiles, particularly involving HIF-1 signaling, amino acid biosynthesis, and AMPK signaling. These pathways play important roles in regulating skin and HF development. The identified genes and metabolites provide potential molecular markers for improving rabbit fur quality and enhance the understanding of HF biology at the molecular level.

## Data Availability

The raw sequencing data have been deposited in the CNCB under accession number OMIX017024 and CRA043744. All datasets used and analyzed during the current study are available from the corresponding author on reasonable request.

## Supplementary Materials

The following supporting information can be downloaded at https://www.mdpi.com/article/10.3390/ani16172746/s1. Table S1. List of primers used for RT-qPCR analysis. Table S2. Differential metabolites identified in the longissimus dorsi muscle between New Zealand White rabbits and Rex rabbits. Table S3. Quality control and mapping statistics of transcriptome sequencing data. Table S4. DEGs identified in dorsal skin tissues between NZW and RX rabbits.
